# Die Videosprechstunde in einer unfallchirurgisch/orthopädischen Spezialsprechstunde: Effektive ergänzende Maßnahme in Lockdown- und Post-Lockdown-Szenarien – eine prospektive Pilotstudie

**DOI:** 10.1007/s00113-021-01032-4

**Published:** 2021-06-29

**Authors:** Jan Theopold, Georg Osterhoff, Peter Melcher, Ralf Henkelmann, Pierre Hepp

**Affiliations:** Klinik für Orthopädie, Unfallchirurgie und Plastische Chirurgie, Bereich für arthroskopische und spezielle Gelenkchirurgie/ Sportverletzungen, Universitätsklinikum Leipzig, Liebigstraße 20, 04103 Leipzig, Deutschland

**Keywords:** Videosprechstunde, Telemedizin, COVID-19, Klinische Untersuchung, Digitalisierung, Video consultation, Telemedicine, COVID-19, Clinical examination, Digitalisation

## Abstract

**Hintergrund:**

Im Rahmen der Kontaktbeschränkungen aufgrund der COVID-19-Pandemie vom März 2020 wurde zur Aufrechterhaltung der Patientenversorgung eine videobasierte Sprechstunde eingeführt. Als Basis einer kontaktminimierenden Kommunikation wurde diese nach den Maßnahmen fortgeführt.

**Ziel der Arbeit:**

Ziel dieser prospektiven Pilotstudie ist die Beurteilung hinsichtlich der Effektivität, der technischen Machbarkeit sowie der Steuerung von Patientenströmen sowohl unter Lockdown-Bedingungen sowie in der Zeit danach.

**Material und Methoden:**

Die Auswertung erfolgte vom Beginn des ersten Lockdowns am 16.03.2020 bis zum Stichtag der zweiten einschränkenden Maßnahmen am 14.12.2020. Dokumentiert wurde die Qualität der Verbindungen bezüglich Ton und Bild. Weiterhin wurden die Konsequenzen aus den Gesprächen dokumentiert. Unterschieden wurde hierbei in 4 Kategorien: 1. keine erneute Vorstellung, 2. Wiedervorstellung in der Videosprechstunde, 3. operative Therapie und 4. Vorstellung zur klinischen Untersuchung.

**Ergebnisse:**

Es erfolgten 236 Patientenvorstellungen mittels Videosprechstunde. Insgesamt erfolgten 182 (82 %) Gespräche ohne Einschränkungen. Bei 47 (21 %) Konsultationen handelte es sich um Erstvorstellungen. Bei 41 (18 %) Patienten erfolgte keine erneute Vorstellung. Bei 36 (16 %) Patienten wurde eine Wiedervorstellung in der Videosprechstunde geplant, bei 36 (16 %) Patienten erfolgte die direkte Einweisung zur Operation, und bei 105 (47 %) Patienten wurde eine Wiedervorstellung zur klinischen Untersuchung vereinbart.

**Diskussion:**

Bei 40 % der Patienten konnte durch den Kontakt in der Videosprechstunde eine definitive Entscheidung gestellt werden. Auf der anderen Seite erfolgte bei 47 % der Patienten eine Vorstellung zur klinischen Untersuchung. Die Videosprechstunde ist eine sehr nützliche Maßnahme, um Patientenaufkommen zu leiten und den direkten Arzt-Patient-Kontakt sichtbar zu unterstützten.

## Abstract (en)

**Background:**

With the regulation of the Saxon State Government and the Saxon State Ministry for Social Affairs and Social Responsibility on the modification of the Infection Protection Act of March 2020 coming into force, a video-based outpatient consultation was implemented to maintain patient care. In order to allow communication with minimized contact, this was continued after the lockdown.

**Aim of the work:**

The aim of this prospective pilot study was to assess the effectiveness of a video-based outpatient consultation service, technical feasibility and control of patient flow under both lockdown and post-lockdown conditions.

**Material and methods:**

The initial evaluation was conducted up to 14 December 2020 when the second restrictive measures were implemented by the state government. The quality of the connections regarding sound and image was documented. Furthermore, the consequences of the conversations were documented. Distinctions were made in four categories: 1. no follow-up visit, 2. follow-up via video consultation, 3. operative intervention and 4. in-person follow-up visit for clinical examination.

**Results:**

There were 236 video-based outpatient consultations, 182 (82%) consultations were without restrictions and 47 (21%) consultations were initial presentations. There were no follow-up consultations in 41 (18%) patients. Video-based follow-up was scheduled in 36 (16%) patients, direct referral for surgery in 36 (16%) patients, and in-person follow-up in 105 (47%) patients.

**Discussion:**

In 40% of the patients a definite decision could be made by the initial video-based consultation alone. On the other hand, 47% of the patients needed in-person follow-up for a clinical examination. Thus, video consultation is a very useful measure to manage patient volume and visibly support direct doctor-patient contact.

## Hintergrund und Fragestellung

Im Rahmen der COVID-19-Pandemie wurden, im Sinne des Infektionsschutzgesetzes, kontaktminimierende Maßnahmen beschlossen [11, 20, 21]. Dies betraf auch Krankenhäuser, um Kapazitäten für infizierte Patienten zu schaffen [1, 10, 21]. Mit Beginn der Kontaktbeschränkung, dem sog. Lockdown im März 2020 [21, 22], wurden auch die ambulanten Behandlungsmöglichkeiten zunehmend eingeschränkt. Entsprechend den Regelungen des American College of Surgeons (ACS) und der Deutschen Gesellschaft für Orthopädie und Unfallchirurgie (DGOU) wurden elektive Eingriffe abgesetzt, um entsprechende Bettenkapazität vorhalten zu können [1, 9, 10]. Auch im Rahmen der Hochschulambulanzen wurde eine Reduktion der Patienten notwendig [12]. Als Alternative boten sich videogestützte Formate, die sog. Videosprechstunden, an. Durch die Politik gefördert, konnten bereits ab 2015 erste Versuche einer Implementierung digitaler Sprechstunden in die Chirurgie erfolgen [6, 7, 29]. Auch in Deutschland erfolgten Vorschläge, eine digitale Sprechstunde in den Praxisalltag zu integrieren [13]. Durch die kassenärztlichen Vereinigungen wurden schließlich 2018 die weiteren Weichen zur Förderung einer digital basierten Vorstellung von Patienten gestellt [3]. Die Videosprechstunde bietet hierbei Flexibilität, erspart Wege und verhindert insbesondere in Zeiten der Pandemie einen direkten Arzt-Patient-Kontakt mit erhöhtem Infektionsrisiko [26]. Zwar fehlen bei einer virtuellen Konsultation die spezifischen klinischen orthopädischen Tests als wesentliches Element der klinischen Untersuchung, aber eine inspektorische Erfassung der Gelenkbeweglichkeit und eine Selbstpalpation des Gelenkes durch den Patienten sind möglich [4]. In Studien wurde auf die Gleichwertigkeit der virtuellen Sprechstunde mit der realen Sprechstunde unter gewissen Voraussetzungen hingewiesen [16, 24]. Unmittelbar mit Beginn des ersten Lockdowns der Coronakrise am 16.03.2020 erfolgte im Bereich für arthroskopische und spezielle Gelenkchirurgie/Sportverletzungen der Klinik für Orthopädie, Unfallchirurgie und Plastische Chirurgie am Universitätsklinikum Leipzig die Umstellung auf eine internetbasierte Videosprechstunde. Auch nach Beendigung der Maßnahmen wurde das Angebot der Videosprechstunde weitergeführt.

Ziel der folgenden prospektiven Pilotstudie ist die Darstellung und Beurteilung der Effektivität einer videobasierten Sprechstunde bezüglich Akzeptanz, technischer Machbarkeit und Performance sowie bezüglich Steuerung von Patientenströmen sowohl unter Lockdown-Bedingungen sowie in der Zeit danach.

## Studiendesign und Untersuchungsmethoden

**Abb. 1:**
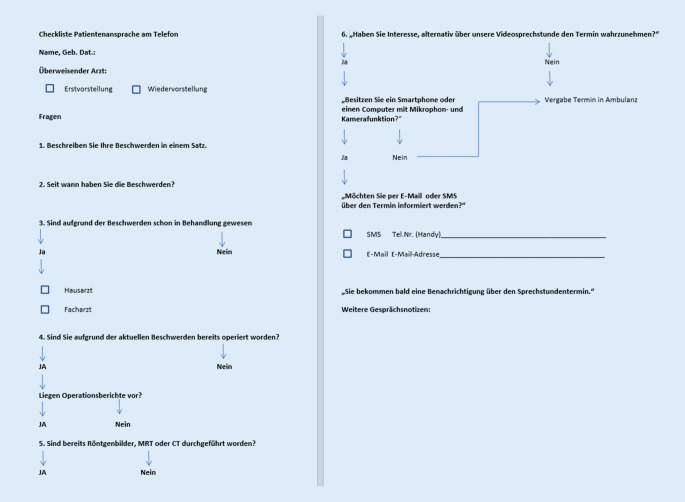
Spezifischer Algorithmus für die Videosprechstunde. Das Sprechstundenpersonal geht bei Terminanfragen den dargestellten Algorithmus mit dem Patienten durch. Bei den *Punkten* *1–5* geht es um die Präzisierung der Beschwerden und der bereits durchgeführten Diagnostik. Ein „Nein“ stellt hierbei kein Abbrechen des Algorithmus dar. Unter *Punkt* *6* ergibt sich eine Eignung für oder gegen die Videosprechstunde

Seit dem 13.03.2020 erfolgte die Onlinesprechstunde als freiwilliges Angebot für die Patienten. Die hier vorgestellte Gelenkspezialsprechstunde findet einmal wöchentlich statt. Die Auswertung erfolgte bis zum Stichtag der einschränkenden Maßnahmen im Rahmen der zweiten Welle der Coronapandemie am 14.12.2020. Im hier vorgestellten Zeitraum wurden 2 Anbieter für videobasierte Sprechstunden genutzt. Initial erfolgte die Sprechstunde mit „Sprechstunde.online“ (Fa. Zava Sprechstunde Online GmbH, Essen, Deutschland). Im weiteren Verlauf erfolgte ein Anbieterwechsel zu „Samedi.de“ (Fa. Samedi GmbH, Berlin). Parallel zur Erfassung klinisch-inspektorischer Befunde und Bewegungsausmaße in der Patientenakte erfolgte die prospektive Dokumentation von Parametern zur Bestimmung der Qualität der Sprechstunde. Die Patienten wurden vorab nach telefonischer Terminabstimmung gemäß einem spezifischen Algorithmus für die Videosprechstunde terminiert (Abb. 1). Die Datenerhebung erfolgte auf Basis des § 34 des Sächsischen Krankenhausgesetzes. Bei Zustandekommen des Termins wurden die Dauer des einzelnen Gesprächs sowie die Qualität des Bildes und des Tones mittels dichotomer Fragen dokumentiert. Weiterhin wurden der Grund der Vorstellung erfasst, ob es sich um eine Erst- oder eine Wiedervorstellung handelte, und die diagnostisch-therapeutischen Konsequenzen der Videokonsultation dokumentiert. Hierbei wurden folgende 4 Kategorien zusammengefasst:

1. Wiedervorstellung des Patienten nur bei Bedarf empfohlen, bzw. es wurde eine Weiterbehandlung durch einen niedergelassenen Kollegen initiiert.
2. Erneute Vorstellung zur Verlaufskontrolle in der Videosprechstunde indiziert.
3. Eine Einweisung zur Operation erfolgte bei eindeutigen Befunden und vollständiger Bildgebung. Hier wurden die Patienten am Tag vor der eigentlichen Operation in personam einbestellt, klinisch vollständig untersucht und die Indikation zur Operation letztmalig geprüft.
4. Vorstellung in der realen Sprechstunde zur exakteren klinischen Evaluation bei unklaren Befunden oder zur Durchführung von Röntgenbildern.

## Ergebnisse

**Abb. 2:**
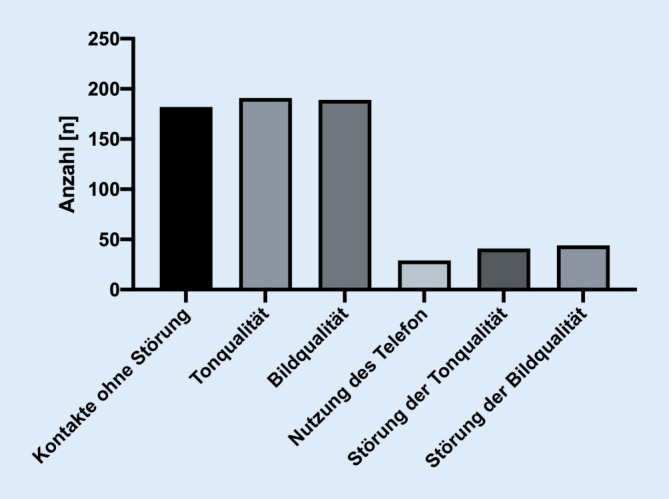
Qualität der Kommunikation

**Abb. 3:**
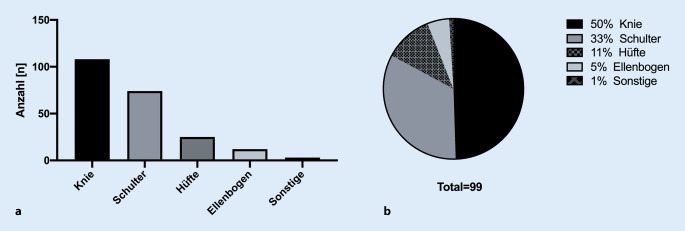
Betroffene Gelenke. Darstellung der Häufigkeit der betroffenen Gelenke/Regionen (**a** absolut; **b** prozentual)

Insgesamt erfolgten im oben genannten Zeitraum 236 videobasierte Patientenvorstellungen. Das Durchschnittsalter der Teilnehmer betrug 37 Jahre (Min. 15, Max. 70), 92 (39 %) waren weiblich. Vierzehn (6 %) der ursprünglich terminierten Patienten konnten weder per Videosprechstunde noch telefonisch erreicht werden und nahmen nicht an der Sprechstunde teil. Insgesamt konnten von allen durchgeführten Gesprächen 182 (82 %) ohne Einschränkungen durchgeführt werden (Abb. 2). Die durchschnittliche reine Gesprächszeit betrug 8:27 min (Min. 1:20; Max. 23:21). Bei 6 (3 %) Kontakten gab es Einschränkungen in der Qualität des Videos, bei 9 (4 %) eine geminderte Qualität des Tones. Bei 29 (13 %) Vorstellungen musste die Konsultation mittels eines Telefons durchgeführt werden, da ein internetbasierter Kontakt nicht möglich bzw. die Videoqualität nicht ausreichend war. Bei 47 (21 %) Konsultationen handelte es sich um Erstvorstellungen und bei 175 (79 %) um eine Wiedervorstellung nach stationärem Aufenthalt oder ambulanter Vorbehandlung. Das am häufigsten betroffene Gelenk bei der Vorstellung in der Videosprechstunde war mit 108 (49 %) das Kniegelenk (Abb. 3). Bei 74 (33 %) Vorstellungen handelte es sich um Beschwerden im Bereich der Schulter. Bei 25 (11 %) Konsultationen war das Hüftgelenk und bei 12 (5 %) der Ellenbogen betroffen. Bei 3 (1 %) Patienten handelte es sich um proximale Ausrisse der Hamstring-Sehnen bzw. um einen Einriss der Peronäalsehne.

**Abb. 4:**
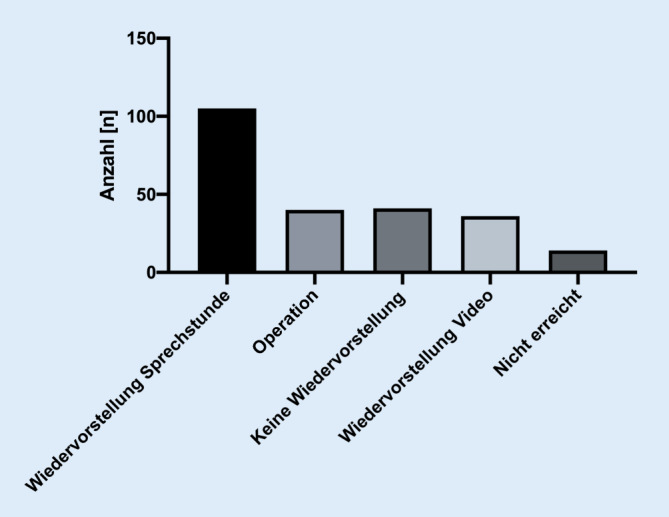
Konsequenzen aus der Konsultation

Als Konsequenz wurde bei 41 (18 %) Patienten eine erneute Vorstellung bei Bedarf bzw. eine Weiterbehandlung durch einen niedergelassenen Kollegen vereinbart (Abb. 4). In 36 (16 %) der Fälle wurde eine erneute Wiedervorstellung zur Verlaufskontrolle in der Videosprechstunde geplant. Bei 105 (47 %) Patienten erfolgte die Wiedervorstellung zur besseren Diagnosefindung in der realen ambulanten Sprechstunde zur Verifizierung der klinischen Befunde. Insgesamt konnte bei 36 (16 %) der Patienten aufgrund der vorhandenen Vorbefunde und der eindeutigen Klinik eine direkte Einweisung zur Operation erfolgen. Hierbei waren bei 20 (56 %) der Patienten Beschwerden an den Kniegelenken führend. Bei 12 (33 %) waren Beschwerden an der Schulter führend, und bei 4 (11 %) erfolgte die Indikationsstellung zur Operation aufgrund von Beschwerden in der Hüfte.

## Diskussion

In Deutschland wird die Zahl der Arztbesuche zunehmend kritisch diskutiert [27]. Hierbei erfolgen durchschnittlich 10 Arztbesuche pro Patient und Jahr [15]. In Schweden etwa werden zur Reduktion der Arztbesuche vermehrt E‑Health-Lösungen verwendet [15]. In den USA wurden für orthopädische Sprechstunden in Zeiten von COVID-19 spezielle Fragebogen zur Untersuchung der Patienten mittels Videosprechstunde entwickelt [17, 18, 25]. In Deutschland hat selbst im Rahmen der Coronapandemie das Angebot von Onlinesprechstunden wie Chats oder Videosprechstunden zu Beginn nur gering zugenommen [19].

Im Verlauf der Pandemie wurden auch in Deutschland zunehmend Formate und Untersuchungstechniken evaluiert und entwickelt [23, 28]. Buvik et al. konnten zeigen, dass die Videosprechstunde von norwegischen Orthopäden als gleichwertig mit der normalen Sprechstunde angesehen wird [7, 8].

Die hier vorgestellte Patientenzahl zeigt eine gute Akzeptanz von Angeboten wie der Videosprechstunde. Das gesamte Kollektiv der hier gewählten Sprechstunde ist mit einem Durchschnittsalter von 37 Jahren ein jüngeres Kollektiv und im Umgang mit modernen Techniken versiert und aufgeschlossen. Aber auch ältere Patienten sind der Integration von modernen Kommunikationsmitteln nicht unaufgeschlossen [24]. So zeigt die hier vorliegende Untersuchung, dass auch Patienten mit höherem Alter ein Interesse an der digitalen Sprechstunde haben.

Mit einer durchschnittlichen Behandlungszeit (Patient-Arzt-Gespräch) von 8 min zeigt sich kein großer Unterschied zu der durchschnittlichen Behandlungszeit eines deutschen Hausarztes [14, 30]. Hierbei ist kritisch anzumerken, dass weder die Vorbereitung noch die Nachbereitung samt Dokumentation berücksichtigt wurden. Insgesamt kann zumindest für den Arzt nicht von einer Verkürzung der aufzuwendenden Zeit pro Patient ausgegangen werden.

Negativ anzumerken ist die aktuell immer noch fehlende Regelung bei der Kostenübernahme insbesondere für Hochschulambulanzen [2]. Dies betrifft u. a. die Ausstellung von Verordnungen bei konservativer Therapie.

Durch die mittlerweile verbesserte Netzabdeckung ist im vorliegenden Set-up eine ausreichende Netzgeschwindigkeit vorhanden, sodass auch in einem relativen Flächenland wie Sachsen eine ausreichende Qualität der Infrastruktur vorliegt, um eine videobasierte Sprechstunde durchzuführen [5]. Auch bei schlechter Bildqualität war teilweise eine weitere Sprechstunde nur mittels Tonübertragung möglich. Nur bei schlechter Tonqualität musste auf eine telefonische Beratung zurückgegriffen werden.

In 21 % der Vorstellungen handelte es sich um Erstvorstellungen. Hierbei können insbesondere extern einzuholende Befunde wie die Schnittbildgebung oder konsiliarische Untersuchungen (z. B. ENG/EMG) auf Vollständigkeit überprüft und ggf. indiziert werden. So lässt sich sicherlich auch die hohe Anzahl an Wiedervorstellungen in der ambulanten Sprechstunde erklären. Ein wesentlicher Vorteil der Videosprechstunde besteht darin, dass die Zahl der Patienten mit unvollständiger Diagnostik in der realen Sprechstunde minimiert werden kann und so Mehrfachvorstellungen vermeiden werden.

Bei 40 % der Patienten konnte bereits in der Videosprechstunde eine definitive Entscheidung zum weiteren Prozedere gestellt werden. Entweder erfolgte die Einleitung einer konservativen funktionellen Therapie oder eine Indikation zur Operation.

Bei der hier vorgestellten Arbeit handelt es sich um eine prospektive reine Beobachtungsstudie ohne Intervention oder Kontrollgruppe. Eine Qualitätsbeurteilung der Bild- und Tonqualität erfolgte nur anhand dichotomer Fragen. Eine differenzierte quantitative Aussage zur Qualität ist somit nicht möglich. Jedoch lässt sich feststellen, dass bei stabiler Verbindung die Qualität der Sprechstunde ausreichend war, um eine adäquate Anamnese durchzuführen. Weiterhin ist anzumerken, dass durch die anonymisierte Datenerhebung eine Mehrfachvorstellung einzelner Patienten nicht ausgeschlossen werden kann.

## Fazit für die Praxis

Zusammenfassend lässt sich feststellen, dass die Videosprechstunde im Lockdown und auch in der Zeit danach ein effektives Verfahren bezüglich Akzeptanz sowie technischer Machbarkeit und Perfomance ist. Die vom Arzt aufzuwendende Zeit wird nicht reduziert. Das Patientenaufkommen kann insofern gesteuert werden, dass Mehrfachvorstellungen aufgrund fehlender Vorbefunde sowie routinemäßige Kontrolluntersuchungen reduziert werden können. Die Videosprechstunde ist somit als ergänzende Maßnahme einzuschätzen, die den direkten Arzt-Patient-Kontakt mit der klinischen Untersuchung jedoch kaum ersetzt.

Aufgrund der positiven Ergebnisse und der hohen Akzeptanz der Videosprechstunde durch die Patienten, insbesondere aus entfernteren Regionen, wird die Videosprechstunde auch nach Pandemiezeiten in unserer Klinik weiterhin angeboten werden.
